# Two-Layer Elastographic 3-D Traction Force Microscopy

**DOI:** 10.1038/srep39315

**Published:** 2017-01-11

**Authors:** Begoña Álvarez-González, Shun Zhang, Manuel Gómez-González, Ruedi Meili, Richard A. Firtel, Juan C. Lasheras, Juan C. del Álamo

**Affiliations:** 1 Division of Cell and Developmental Biology, University of California, San Diego; 2 Department of Mechanical and Aerospace Engineeing, University of California, San Diego; 3 Department of Bioengineering, University of California, San Diego; 4 Center for Medical Devices and Instrumentation, Institute for Engineering in Medicine, University of California, San Diego.

## Abstract

Cellular traction force microscopy (TFM) requires knowledge of the mechanical properties of the substratum where the cells adhere to calculate cell-generated forces from measurements of substratum deformation. Polymer-based hydrogels are broadly used for TFM due to their linearly elastic behavior in the range of measured deformations. However, the calculated stresses, particularly their spatial patterns, can be highly sensitive to the substratum’s Poisson’s ratio. We present two-layer elastographic TFM (2LETFM), a method that allows for simultaneously measuring the Poisson’s ratio of the substratum while also determining the cell-generated forces. The new method exploits the analytical solution of the elastostatic equation and deformation measurements from two layers of the substratum. We perform an in silico analysis of 2LETFM concluding that this technique is robust with respect to TFM experimental parameters, and remains accurate even for noisy measurement data. We also provide experimental proof of principle of 2LETFM by simultaneously measuring the stresses exerted by migrating Physarum amoeboae on the surface of polyacrylamide substrata, and the Poisson’s ratio of the substrata. The 2LETFM method could be generalized to concurrently determine the mechanical properties and cell-generated forces in more physiologically relevant extracellular environments, opening new possibilities to study cell-matrix interactions.

The mechanical properties of the extracellular environment affect cellular behavior and processes such as cell migration, proliferation, growth, differentiation, and spreading^1^,^2^,^3^. Cells can feel the mechanical properties of their extracellular environment and regulate their adhesions by a process known as mechanosensing^4^,^5^,^6^. Traction forces exerted by the cells are known to regulate not only cell locomotion but also many other cellular processes^7^,^8^. Several traction force microscopy methods have been developed to measure the forces exerted by stationary and/or migrating cells on flat elastic polymer-based hydrogels^9^,^10^,^11^,^12^,^13^,^14^,^15^. These gels exhibit a linearly elastic behavior in the range of the small deformations produced by the cells^16^,^17^,^18^. The calculation of the traction forces in these TFM methods requires a precise knowledge of the constitutive equations of the substratum, which for linearly elastic materials depend only on two parameters: the Young’s modulus of elasticity and the Poisson’s ratio^19^. The Young’s modulus of polyacrylamide and other elastic materials commonly used in these TFM methods are well-known, and there are established methods for their measurement^17^. On the other hand, the value of their Poisson’s ratio is often not well characterized.

Flexible polymer hydrogels have been shown to exhibit Poisson’s ratios close to 0.5. However, a wide range of values has been reported for these gels in the literature (0.27–0.49), depending on their specific composition and method of preparation^20^,^21^,^22^. Pioneering TFM studies that assumed 2-D substratum deformation and infinite substratum thickness reported a weak dependence of the traction stresses on the Poisson’s ratio^23^. However, more recent analyses that consider the finite thickness of the substratum and three-dimensional deformations have indicated that this dependence is stronger than previously believed^24^. The uncertainty in the Poisson’s ratio poses an important limitation to TFM methods since for a given deformation field, not only the magnitude but also the spatial distribution of the traction forces depends on the Poisson’s ratio^25^. To address this issue, we have developed a new traction force microscopy method that enables the simultaneous calculation of the Poisson’s ratio of the gel and the traction forces that a cell exerts on it. Furthermore, this method allows for measuring the Poisson’s ratio at each specific measurement time and location, in order to account for possible spatial and temporal variations of the mechanical properties of the substratum when measuring cellular traction forces, cell-cell tensions^25^,^26^, and potentially other biomechanical quantities of interest.

When cells adhere to an elastic substratum, they apply forces on its surface producing deformations deep throughout the substratum^27^. These deformations depend on the value of the Poisson’s ratio of the substratum’s material, *σ*^24^. If the Poisson’s ratio is known, it is possible to solve the elastostatic equation


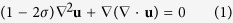


to determine the 3-D deformation everywhere inside the substratum, **u**(*x, y, z*), from the 3-D deformation measured on a single plane, **u**(*x, y, z* = constant)^24^. Thus, knowing the 3-D substratum deformation on two different planes, **u**(*x, y, z* = *h*_0_) and **u**(*x, y, z* = *h*_1_), makes equation (1) highly overdetermined. In this paper, we exploit this overdetermination to develop a two-layer elastographic traction force microscopy (2LETFM) method that allows us to estimate *σ* while simultaneously calculating the traction forces exerted by the cell. It is important to note, however, that this method cannot determine the Young’s modulus of the substratum because this parameter modulates the deformations in the same manner everywhere in the substratum and, thus, it does not affect the deformation patterns.

2LETFM is rooted in the analytical solution to the elastostatic equation (1) developed for Fourier Traction Force Microscopy^10^,^24^,^28^. We use this solution together with the deformation measured at the first plane (*z* = *h*_0_) to calculate the deformation on the second plane (*z* = *h*_1_) as a function of the Poisson’s ratio. Then, we determine the Poisson’s ratio by iteratively minimizing the least-squares error between the measured and calculated deformations at *z* = *h*_1_. The obtained value of *σ* is subsequently used to calculate the 3-D traction stresses exerted by the cell following the approach described by del Álamo *et al*.^24^. To test the accuracy and robustness of the new 2LETFM method, we use synthetic deformation fields with prescribed background noise. This analysis indicates that, in the range of values of the Poisson’s ratio typically encountered in experiments (*σ* > 0.3), 2LETFM can accurately and robustly determine *σ* for a wide range of experimental design parameters, and even in the presence of significant measurement noise. As way of illustration, we perform 2LETFM experiments on *Physarum* microamoebae migrating on the flat surface of polyacrylamide substrata. The elastographic TFM analysis can be immediately extended to the case of cells embedded inside linearly elastic 3-D matrices.

## Methods

### Two-layer elastographic traction force microscopy analysis

**Figure 1:**
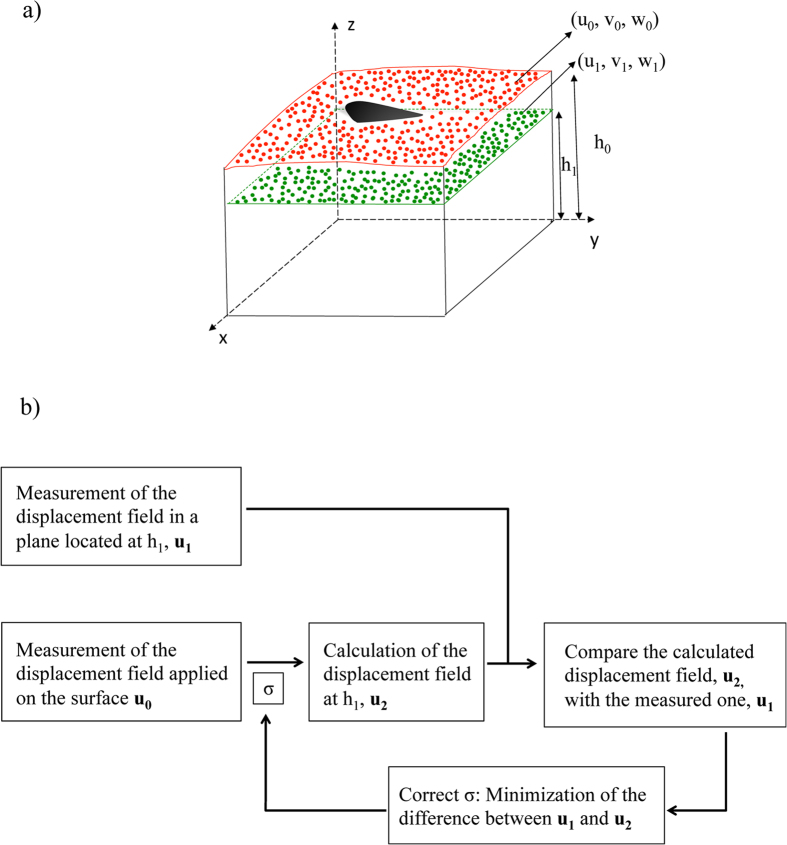
(**a**) Sketch of the configuration of the experiment. The substratum has a layer embedded with red beads at the surface and another layer embedded with green beads in a plane below the surface. As a cell adheres to the substratum, the deformations that it applies in both layers embedded with beads are measured. (**b**) Overview of the method. Diagram indicating the steps followed in this method for the calculation of the Poisson’s ratio of the substratum.

Consider the two-layer TFM setup in Fig. 1a, where the 3-D deformation of the substratum is measured at two separate horizontal planes, **u**_0_ = **u**(*x, y, z* = *h*_0_) and **u**_1_ = **u**(*x, y, z* = *h*_1_). For this exposition, we will consider that *h* ≥ *h*_0_ > *h*_1_ where *h* is the substratum thickness. Using **u**_0_ as boundary condition together with zero deformation at the bottom of the substratum, it is possible to find an exact solution to the elastostatic equation (1) where the Poisson’s ratio is a free parameter^24^. This solution provides the full 3-D deformation vector field everywhere inside the substratum, including at the second measurement plane *z* = *h*_1_.

The solution procedure, which was explained in detail by del Álamo *et al*.^24^,^28^, is summarized as follows. We expand the deformation vector field in Fourier series in the *x* and *y* directions,


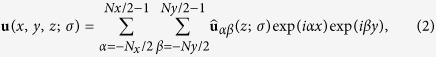


where *N*_*x*_ and *N*_*y*_ are respectively the number of measurement points in *x* and *y*, and *α* and *β* are the corresponding wavenumbers. This transformation allows us to obtain a second-order, ordinary boundary value problem for 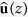. The solution to this problem is


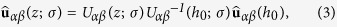


where *U*_*αβ*_(*z*; *σ*) is the resolvant matrix of the boundary value problem, which can be found in closed analytical form in ref. 24, and 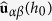 are the Fourier coefficients of the deformation measured at *z* = *h*_0_. By plugging *z* = *h*_1_ in equations (2, 3), we calculate the substratum deformation at the second measurement plane from the substratum deformation measured at the first plane,


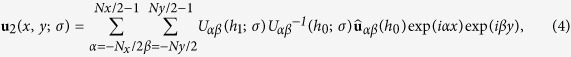


which is equivalent to 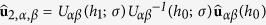. The Poisson’s ratio appears as a free parameter in the calculation of **u**_2_. Thus, comparing **u**_2_(*x, y*; *σ*) with the deformations measured at the second plane, **u**_**1**_(*x, y*), allows for estimating the value of the Poisson’s ratio *σ*_*r*_ that maximizes their agreement.

The resolvant matrix of the traction force microscopy problem has a form that can amplify or suppress experimental noise^28^,^29^. Specifically, it is straightforward to see that deformations with spatial patterns of wavelength 
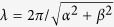
 much smaller than substratum thickness (*λ* ≪ *h*) vary as as 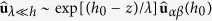
28. This dependence justifies our choice *h*_0_ > *h*_1_, which naturally introduces a low-pass filter for the noise contained in the deformations measured at *z* = *h*_0_. Note that the alternative option, *h*_0_ < *h*_1_, would exponentially amplify measurement noise. Experimental errors can be decreased further given that the comparison *u*_2_(*x, y*; *σ*) = *u*_1_(*x, y*) is done at *N*_*x*_ × *N*_*y*_ different points (see eq. 4). Having *N*_*x*_ × *N*_*y*_ conditions to estimate one single parameter makes the calculation of *σ*_*r*_ highly overdetermined and renders the error of the recovered Poisson’s ratio significantly lower than that of the traction stresses themselves, as is shown below. To solve the overdetermined problem, we followed a least-squares approach using an iterative Levenberg-Marquardt algorithm that minimized a global cost function to calculate *σ*. This iterative algorithm is similar to those employed in our previous studies^30^,^31^,^32^ and is summarized in Fig. 1b.

We tested two different cost functions,


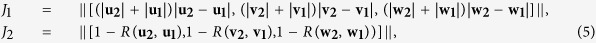


where *R* stands for the correlation coefficient. The main difference between these two functions is that *J*_1_ penalizes differences in magnitude whereas *J*_2_ penalizes differences in spatial patterns.

Once the value of *σ*_*r*_ has been estimated, the traction stresses at the cell-substratum interface are obtained as described by del Álamo *et al*.^24^. Specifically, these stresses are determined from the calculated displacements and their *z*-derivatives by applying Hooke’s law in Fourier space,


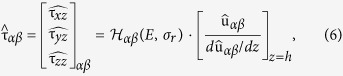


where the 6 × 3 matrix 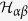, which only depends on the material properties of the substratum (*E* and *σ*_*r*_) and the wavenumbers of each Fourier mode, was given in the Supporting Information of del Álamo *et al*.^24^.

### Cell culture

We cultured *Physarum polycephalum* plasmodia in agarose plates with oatmeal^33^,^34^. We excised small pieces from the parent plasmodium, and grew them on agarose plates without oatmeal during 10 hours. We subsequently excised 200-*μ*m sized pieces with a scalpel and placed them over the polyacrylamide gel. This process produces highly motile amoebae^35^,^36^. We then removed the surrounding water using a small glass capillary tube, and covered the polyacrylamide gel with the amoebae using an agar cap. To fabricate the agar cap, we boiled a 100 mM agar solution and poured 100 *μ*l of this solution on a glass coverslip mounted with a 12-mm washer, and waited until the agar solidified. The weight of the agar cap generated a pressure on the amoebae comparable to but smaller than the traction stresses generated by the cells, thus generating a gentle confinement^36^. Such confinement prevented the gel from drying out and rendered the cell easier to visualize, while still allowing the cell to exert significant deformations in the substratum.

### Substratum fabrication

We fabricated 12-mm diameter polyacrylamide gels of 5% acrylamide and 0.225% bisacrylamide (Young’s modulus approximately 8 KPa^37^) mounted on 22-mm square # 1 glass coverslips^38^,^39^. To independently measure the substratum deformation at a plane near its surface (*z* = *h*_0_) and at a second plane below the surface (*z* = *h*_1_), we fabricated a polyacrylamide substratum with four layers: the bottom layer contained no beads, the second layer contained 0.07% carboxylate modified yellow latex beads of 0.5 *μ*m diameter (Fluospheres, Invitrogen, Carlsbad CA), the third layer contained no beads, and the top layer contained 0.07% carboxylate modified red latex beads of 0.5 *μ*m diameter (Fluospheres, Invitrogen, Carlsbad CA). Figure 1a shows a sketch of the experimental configuration. The layers were verified to adhere well to each other under experimental conditions by confirming continuity of deformation across different layers, similar to our previous work^24^. To further establish if the layers were properly attached to each other so that there were no jumps in deformation across different layers, we verified the continuity of the substratum deformation in *x* − *z* planes under the cells (see Figure S1 in the Supplementary Information). We mounted the coverslips with the gels in Petri dishes with circular openings at their bottom using silicon grease (Dow Corning, Midland, Michigan). We made the gels physiologically compatible by cross-linking collagen I to the surface of the polyacrylamide. We used 1 mM Sulfo-SANPAH (Thermo Sci, Rockford, Il) after UV activation to cross-link 0.25 mg/ml collagen I. The gels were incubated overnight at room temperature. After washing, the gels were stored in 0.05 M HEPES buffer and antibiotic (40 *μ*M Ampicillin) for up to a week. The thickness of the gels was approximately 100 *μ*m and the distance between the two layers containing beads was approximately 15 *μ*m. In each experiment, we measured the exact thickness of the gel and the distance between the two layers containing beads. The thickness of the gel was measured by locating the top and bottom planes and subtracting their *z*-positions and, in a similar manner, the distance between the two layers containing beads was measured by locating these layers and subtracting their *z*-positions.

### Microscopy

The 3-D deformation of the substratum at two different planes beneath the surface, *z* = *h*_0_ and *z* = *h*_1_, was measured by tracking respectively the red and yellow fluorescent beads. We acquired time-lapse sequences of image *z*-stacks using a Leica DMI6000 B inverted microscope (Leica Microsystems, Inc., Buffalo Grove, IL) equipped with a Zyla 4.2 sCMOS camera (Andor Technology Ltd., Belfast, UK) and a 20x objective lens. The imaging setup was controlled by the open source microscopy software Micro-Manager^40^,^41^. Each *z*-stack consisted of 20 images at 0.5 *μ*m increments, and was vertically centered at the plane of maximum fluorescence intensity as determined by the autofocus system. The position and shape of the cell at each instant of time was recorded with an additional bright-field phase contrast image. We acquired the two fluorescent *z*-stacks and the phase-contrast image of the cell every 60 seconds.

### Measurement of substratum deformation

We determined independently the 3-D deformation at the center plane of the two imaged *z*-stacks by analyzing the images of the red and yellow beads separately. For each color, we cross-correlated an instantaneous *z*-stack with the cell in the center of the *x* − *y* field of view, and a reference *z*-stack that was obtained after the highly motile cell had moved away of the field of view, similar to our previous works^8^,^24^,^28^,^36^,^42^,^43^,^44^,^45^,^46^. Enough time was given for the cell to move away from the field of view so that the edges of the reference images were non-deformed. We note that the agar cap was not removed to acquire the reference *z*-stack, and thus the polyacrylamide gel was still supporting the weight of the cap. However, this does not preclude the calculation of the traction stresses because the weight exerted by the agar cap is constant, and the polyacrylamide gel behaves linearly (see detailed demonstration in the Supplementary Information). To measure deformation, both the instantaneous and reference *z*-stacks were divided into smaller 3-D interrogation boxes, as is done in particle image velocimetry^47^. In order to balance spatial resolution and signal-to-noise ratio while minimizing phototoxic effects, we chose interrogation boxes of size 64 × 64 × 20 pixels in the *x, y* and *z* directions, with 50% overlap in the *x* and *y* directions. The resulting spatial resolution (20 *μm*) was sufficient for the *Physarum* amoebae considered in our experiments, which had a typical size of 200-*μ*m.

## Results

### Synthetic-field analysis of 2LETFM

**Figure 2:**
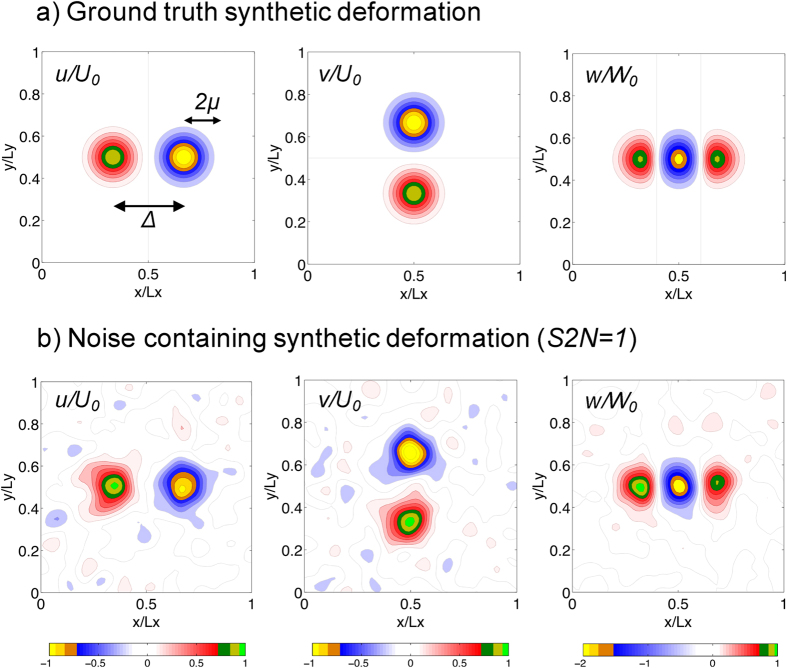
Example of the synthetic deformations used for the *in silico* analysis of 2LETFM. (**a**) Ground truth synthetic deformation, **u**_0_ = (*u*_0_, *v*_0_, *w*_0_) at the surface of the substratum, given by eq. 7. The parameters Δ (distance between positive and negative deformation patterns) and *μ* (size of each the deformation pattern) are explained in the plot. (**b**) Noise containing synthetic field obtained by adding a normally distributed random field that has been low-pass filtered with a Gaussian filter of size *μ*/2. The signal-to-noise ratio of this example is *S*2*N* = 1. The in-plane (*u*_0_, *v*_0_) and out-of-plane (*w*_0_) deformations are given normalized with *U*_0_ and *W*_0_ respectively, and their values correspond to the colorbars at the bottom of each column.

As discussed in the Methods section, the relative position of the measurement planes may influence the performance of 2LETFM in the presence of experimental noise. The Poisson’s ratio and the nature (tangential vs. normal) of the cell-generated traction forces could also affect the accuracy of the new technique, because they modulate how stress and deformation propagate throughout the substratum^24^. To systematically determinate the accuracy and robustness of 2LETFM as a function of these parameters, we applied the new method to synthetic deformation fields with prescribed noise. The synthetic deformation fields were defined by the equations


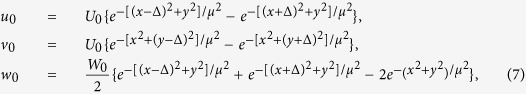


and are shown in Fig. 2. These synthetic data mimic the substratum deformation generated by migrating amoeboid cells^13^, including the *Physarum* amoebae reported in the Results Section below (see also ref. 36). The parameter *μ* in these equations represents the length scale of the deformation patterns, whereas the parameters *U*_0_ and *W*_0_ represent the magnitude of the deformations. To simplify the analysis, we reduced the number of parameters by assuming that the top measurement plane was located at the surface of the substratum, *h* = *h*_0_.

We used the synthetic deformation field **u**_0_ as boundary conditions for the elastostatic equation, and calculated **u**_**1**_(*x, y*) at *z* = *h*_1_ from eq. 4 for given values of the Poisson’s ratio *σ*_*e*_ that was considered as exact. Then, we studied how accurately the two-layer algorithm could recover *σ*_*e*_ under different conditions.

### Effect of Experimental Noise

To model experimental noise, we used **u**_0_ and **u**_**1**_ as ground truth data, and added an independent random field on each of these deformation fields. Noise fields **u**_*nois*_ with spatial patterns that resembled experimental conditions were generated in the Fourier domain, with a Gaussian spectrum of wavenumber width *δ*, i.e.


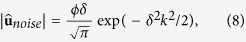


where 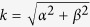, and uniformly random phase. The noise fields generated by this model are also Gaussian in the physical domain (*x, y*), and have a characteristic length scale *δ*. Unless otherwise specified, we used *δ* = *μ*/2 in our simulations so that the length scales of the noise and the ground truth are comparable. Note that this is a conservative scenario that makes it challenging to low-pass or high-pass filter the noise without affecting the ground truth measurements. Additional simulations were performed for other values of *δ*. The results of these simulations, reported in Figure S3 in the Supplementary Information, indicate that the conclusions presented here are independent of *δ*. The S2N of the noise-containing fields was defined as the ratio between the ground truth fields and the root mean squares of the noise,


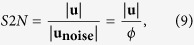


and was varied in the range 0.25 ≤ S2N ≤ 10 via the coefficient *ϕ* in eq. (8). An example of a 3-D noise-containing deformation field with signal-to-noise ratio (S2N) equal to one is shown in Fig. 2b.

We simulated gels with values of the Poisson’s ratio in the range 0.1 < *σ*_*e*_ < 0.5. This range was chosen for completeness, even though values *σ* < 0.3 have not been reported for the elastic hydrogels used in TFM. For each pair of values of *σ*_*e*_ and S2N, we ran *N* = 100 realizations with different random noise distributions. We plugged the noise-containing deformation fields into the least-squares iterative procedure described in Fig. 1 to recover the Poisson’s ratio of the substratum, *σ*_*r*_, and compared the result to *σ*_*e*_. In these simulations, we kept the positions of the two measurement planes fixed at *h*_1_ = 0.85 *h* and *h*_0_ = *h*. The effect of the relative position of the measurement planes, *h*_0_ − *h*_1_ is discussed below.

**Figure 3:**
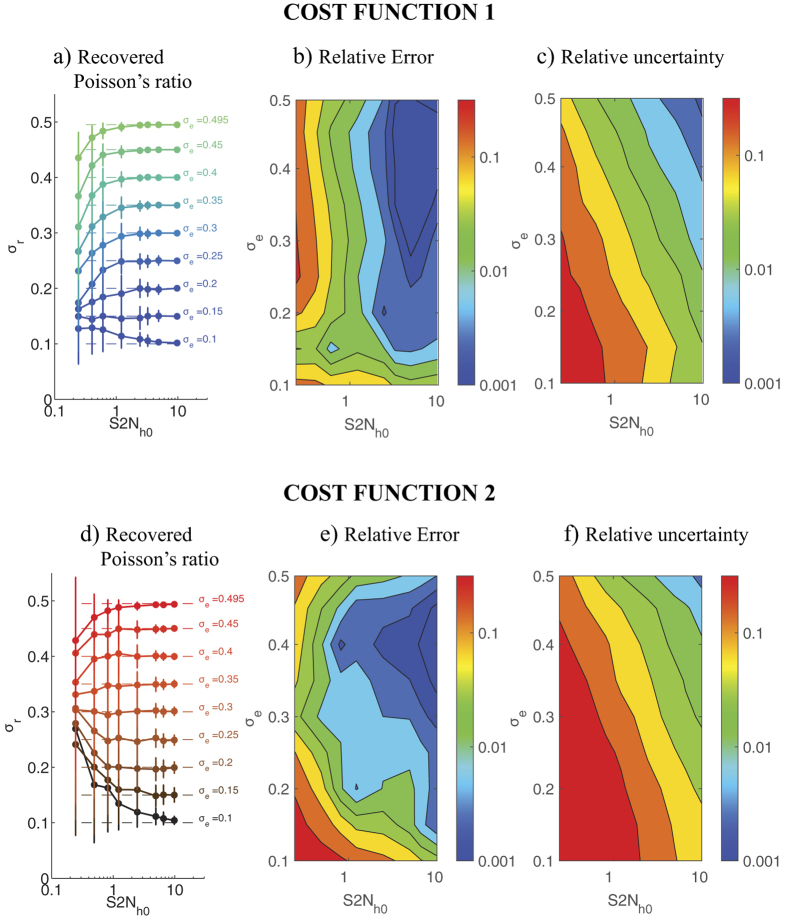
Accuracy and robustness of 2LETFM in the presence of zero-average, normally distributed random noise. (**a**) Value *σ*_*r*_ of the Poisson’s ratio recovered by 2LETFM using the difference cost function *J*_1_ (eq. 5). The data are plotted as a function of the signal-to-noise ratio *S*2*N*_*h*0_ of the deformations prescribed at *z* = *h*_0_, and represented as mean ± standard deviation obtained from *N* = 100 random realizations. Each curve is obtained for a different value *σ*_*e*_ of the exact Poisson’s ratio that is being recovered. This exact value is indicated with a dashed horizontal line in each case. (**b**) Contour plot of the relative error of *σ*_*r*_ obtained with the cost function *J*_1_ as a function of *S*2*N*_*h*0_ and *σ*_*e*_. The relative error is defined as |*σ*_*r*_ − *σ*_*e*_|/*σ*_*e*_. (**c**) Contour plot of the relative uncertainty of *σ*_*r*_ obtained with the cost function *J*_1_ as a function of *S*2*N*_*h*0_ and *σ*_*e*_. The relative uncertainty is defined as r.m.s.(*σ*_*r*_)|/*σ*_*e*_. (**d**) Value *σ*_*r*_ of the Poisson’s ratio recovered by 2LETFM using the correlation cost function *J*_2_ (eq. 5). The data are plotted in the same manner as in panel (**a**). (**e**) Contour plot of the relative error of *σ*_*r*_ obtained with the cost function *J*_2_ as a function of *S*2*N*_*h*0_ and *σ*_*e*_. (**f**) Contour plot of the relative uncertainty of *σ*_*r*_ obtained with the cost function *J*_2_ as a function of *S*2*N*_*h*0_ and *σ*_*e*_. The data were obtained for *h*_0_ − *h*_1_ = 0.15*h*_0_ = 2.5*μ* and *U*_0_ = *W*_0_.

Figure 3 shows the statistics (mean ± standard deviation) of the recovered Poisson’s ratio *σ*_*r*_ as a function of the exact value *σ*_*e*_ and the S2N for the two different cost functions presented in equation 5. The data show that 2LETFM can accurately determine the Poisson’s ratio even for noisy displacement fields. The method is particularly accurate and robust for the values of the Poisson’s ratio typically found in TFM substrata (i.e. 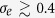). In this range, both |*σ*_*r*_ − *σ*_*e*_| and the standard deviation of *σ*_*r*_ are less than 10% of *σ*_*e*_ for S2Ns as low as 0.5. When the S2N is lower than 0.5, the Poisson’s ratio can be underestimated for *σ*_*e*_ ≥ 0.2 − 0.3, while it is overestimated for lower values of *σ*_*e*_ (Fig. 3a,d). It is worth noting that the determination of *σ* by 2LETFM is substantially more accurate than the calculation of the traction stresses when the same level of noise is present in the measured deformations. In fact, comparing Fig. 3b,e with Figure S3 in the Supplementary Information reveals that the error in *σ*_*r*_ is about 10 times lower than the error in the traction stresses.

The recovery of the Poisson’s ratio is relatively independent of the cost function employed in the least-squares optimization algorithm. However, the cost function based on the difference between deformations (*J*_1_, see eq. 5) (Fig. 3a–c) performs somewhat better than the one based on cross-correlating the spatial patterns of the deformations (*J*_2_, see eq. 5) (Fig. 3d–f), particularly for very low S2Ns. Unless otherwise indicated, the results presented below are obtained using *J*_1._

### Effect of the position of the measurement planes

**Figure 4:**
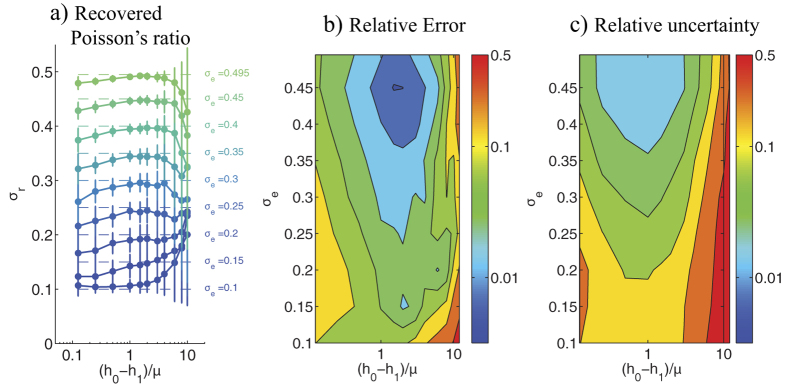
Accuracy and robustness of 2LETFM as a function of the ratio of the distance between the two measurement planes *h*_0_ − *h*_1_ and the length scale of the deformation field at the top plane *μ*. (**a**) Recovered value *σ*_*r*_ of the Poisson’s ratio, plotted as a function of *h*_0_ − *h*_1_. The data are represented as mean ± standard deviation obtained from *N* = 100 random realizations. Each curve is obtained for a different value *σ*_*e*_ of the exact Poisson’s ratio that is being recovered by 2LETFM. This exact value is indicated with a dashed horizontal line in each case. (**b**) Contour plot of the relative error of *σ*_*r*_ as a function of *h*_0_ − *h*_1_ and *σ*_*e*_. The relative error is defined as |*σ*_*r*_ − *σ*_*e*_|/*σ*_*e*_. (**c**) Contour plot of the relative uncertainty of *σ*_*r*_ as a function of *h*_0_ − *h*_1_ and *σ*_*e*_. The relative uncertainty is defined as r.m.s. (*σ*_*r*_)|/*σ*_*e*_. The data were obtained for *S*2*N* = 1 and *U*_0_ = *W*_0_.

The position of the second measurement plane, *z* = *h*_1_, might be a key factor in the experimental design of 2LETFM. To investigate if there is an optimal position that maximizes the accuracy of 2LETFM, we ran simulations varying the value of *h*_1_ and with the noise-containing synthetic fields described above (keeping the S2N = 1). The results of these simulations are summarized in Fig. 4 as a function of *h*_0_ − *h*_1_.

Consistent with the data shown in Fig. 3, the performance of the technique improved as *σ*_*e*_ approached 0.5, both in terms of the average *σ*_*r*_ (Fig. 4a) and its relative error and relative uncertainty (Fig. 4b,c). Furthermore, we found a non-monotonic dependence of these parameters with *h*_0_ − *h*_1_. The algorithm’s performance was optimal when the distance between the two measurement planes, *h*_0_ − *h*_1_ was comparable to the length scale of the deformation patterns at the substratum’s surface, *μ* (see eq. 7). These results can be explained considering that when the second plane is located at a distance from the surface much larger than *μ*, the deformation measured on that plane is negligible^28^. On the other hand, if *h*_1_ ≈ *h*_0_, the deformations measured on the two planes are almost the same. In both cases, the TFM problem is hardly overdetermined and the accuracy of the two-layer approach deteriorates. Despite this non-monotonic behavior, the relative error of the recovered *σ*_*r*_ remains below 10% in a wide range of values of *h*_0_ − *h*_1_.

**Figure 5:**
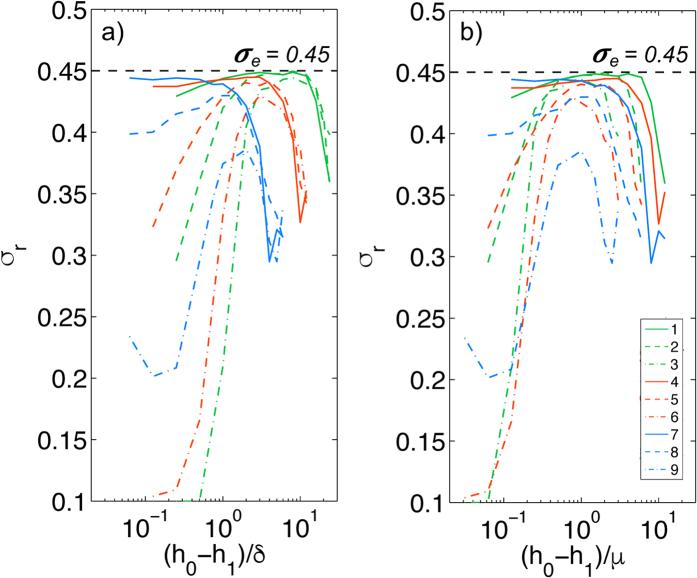
Recovered value *σ*_*r*_ of the Poisson’s ratio, obtained for *σ*_*e*_ = 0.45 and for different values of *μ* and *δ*, and plotted as a function of inter measurement plane distance *h*_0_ − *h*_1_. The legend indicates the different cases considered. Case 1: *μ*/*h*_0_ = 0.02, *δ*/*h*_0_ = 0.01. Case 2: *μ*/*h*_0_ = 0.02, *δ*/*h*_0_ = 0.02. Case 3: *μ*/*h*_0_ = 0.02, *δ*/*h*_0_ = 0.04. Case 4: *μ*/*h*_0_ = 0.04, *δ*/*h*_0_ = 0.01. Case 5: *μ*/*h*_0_ = 0.04, *δ*/*h*_0_ = 0.02. Case 6: *μ*/*h*_0_ = 0.04, *δ*/*h*_0_ = 0.04. Case 7: *μ*/*h*_0_ = 0.08, *δ*/*h*_0_ = 0.01. Case 8: *μ*/*h*_0_ = 0.08, *δ*/*h*_0_ = 0.02. Case 9: *μ*/*h*_0_ = 0.08, *δ*/*h*_0_ = 0.04. (**a**) The inter measurement plane distance *h*_0_ − *h*_1_ is normalized with the length scale of the background noise (*δ*). (**b**) The inter measurement plane distance *h*_0_ − *h*_1_ is normalized with the length scale of the deformation field at the top plane (*μ*). The data were obtained for *S*2*N* = 1 and *U*_0_ = *W*_0_.

In the simulations presented so far, the length scale of the ground-truth synthetic displacements was proportional to that of the noise. Specifically, the 1/*e*-width of the deformation patterns was equal to the root mean square of the ground truth synthetic deformation, *μ* (eq. 7) and the spectral width of the noise distribution was fixed as half that value, *δ* = *μ*/2 (eq. 8). To verify that the optimal inter measurement plane distance is proportional to the length scale of the deformation applied by the cell (*μ*) and not to the length scale of the noise, (*δ*), we performed simulations varying both *δ* and *μ* in addition to *h*_1_. Figure 5 shows the average *σ*_*r*_ as a function of *h*_0_ − *h*_1_ for *σ*_*e*_ = 0.45 for varying the values of *μ* and *δ* in the range 0.01*h*_0_–0.04*h*_0_. The figure shows that when *h*_0_ − *h*_1_ is is normalized with the length scale of the noise patterns (*δ*), *σ*_*r*_ is scattered (Fig. 5a). However, when *h*_0_ − *h*_1_ is normalized with the length scale of the ground-truth deformation patterns (*μ*), the different curves collapse reasonably well. Thus, we conclude that the optimal separation between the measurement planes is associated with the length scale of the cell-generated deformation and not to the measurement noise. Furthermore, the data suggests that *σ*_*r*_ is closest to *σ*_*e*_ for *h*_0_ − *h*_1_ ≈ *μ*.

### Effect of normal/tangential traction force ratio

**Figure 6:**
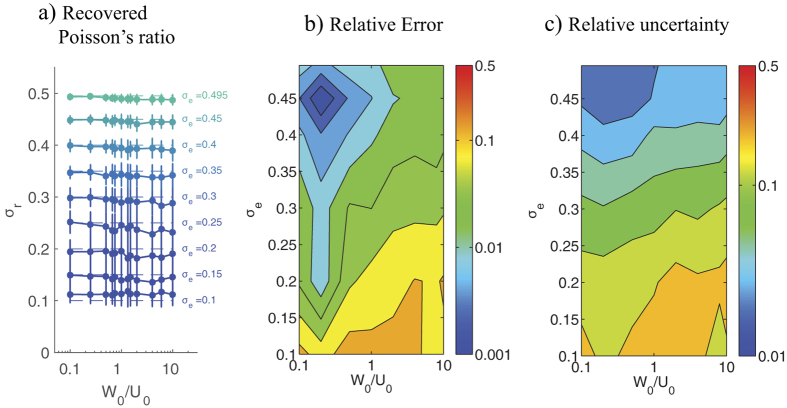
Accuracy and robustness of 2LETFM as a function of the ratio of normal to tangential deformation created by the cell on the substrate, *W*_0_/*U*_0_. (**a**) Recovered value *σ*_*r*_ of the Poisson’s ratio, plotted as a function of *W*_0_/*U*_0_. The data are represented as mean ± standard deviation obtained from *N* = 100 random realizations. Each curve is obtained for a different value *σ*_*e*_ of the exact Poisson’s ratio that is being recovered by 2LETFM. This exact value is indicated with a dashed horizontal line in each case. (**b**) Contour plot of the relative error of *σ*_*r*_ as a function of *W*_0_/*U*_0_ and *σ*_*e*_. The relative error is defined as |*σ*_*r*_ − *σ*_*e*_|/*σ*_*e*_. (**c**) Contour plot of the relative uncertainty of *σ*_*r*_ as a function of *W*_0_/*U*_0_ and *σ*_*e*_. The relative uncertainty is defined as r.m.s. (*σ*_*r*_)|/*σ*_*e*_. The data were obtained for *S*2*N* = 1 and *h*_0_ − *h*_1_ = 0.15*h*_0_ = 2.5 *μ*.

When cells apply traction stresses on the surface of their substratum, the normal component of the cell-generated deformation decays slower with the distance to the surface than the tangential ones^24^. Because 2LETFM relies on detecting differences in the transmission of deformation throughout the substratum, we hypothesized that this technique will be less accurate when cells exert predominantly normal traction stresses in substrata with *σ* ≈ 0.5. To test this hypothesis, we made the synthetic horizontal and vertical deformations using two different length scales, *U*_0_ and *W*_0_ (see eq. 7), and determined the dependence of the error of 2LETFM on the ratio *W*_0_/*U*_0_. The results of this analysis (Fig. 6a–c) confirm our hypothesis, and indicate that the error in the recovered Poisson’s ratio increases with the ratio *W*_0_/*U*_0_. The data also indicates that *σ* can be recovered with reasonable accuracy regardless of *W*_0_/*U*_0_ as long as *σ*_*e*_ > 0.4.

### Experimental demonstration of two-layer elastographic traction force microscopy

To demonstrate the experimental feasibility of 2LETFM, we applied this technique to simultaneously measure the traction stresses generated by migrating *Physarum* amoebae and the Poisson’s ratio of the polyacrylamide substrata where they adhere. For this purpose, we manufactured polyacrylamide gel substrata embedded with fluorescent beads of two different colors in two different layers located at the substratum surface and below it (see Fig. 1). The 3-D substratum deformations in these two layers were independently measured by tracking the displacements of the two sets of fluorescent beads as described in the Methods Section. Using these data, we implemented the two-layer algorithm presented above to estimate the Poisson’s ratio *σ* of the polyacrylamide gel and, once *σ* was known, determined the traction stresses generated by the cell.

### Recovered Poisson’s ratio. Effect on deformations and traction stresses

**Figure 7:**
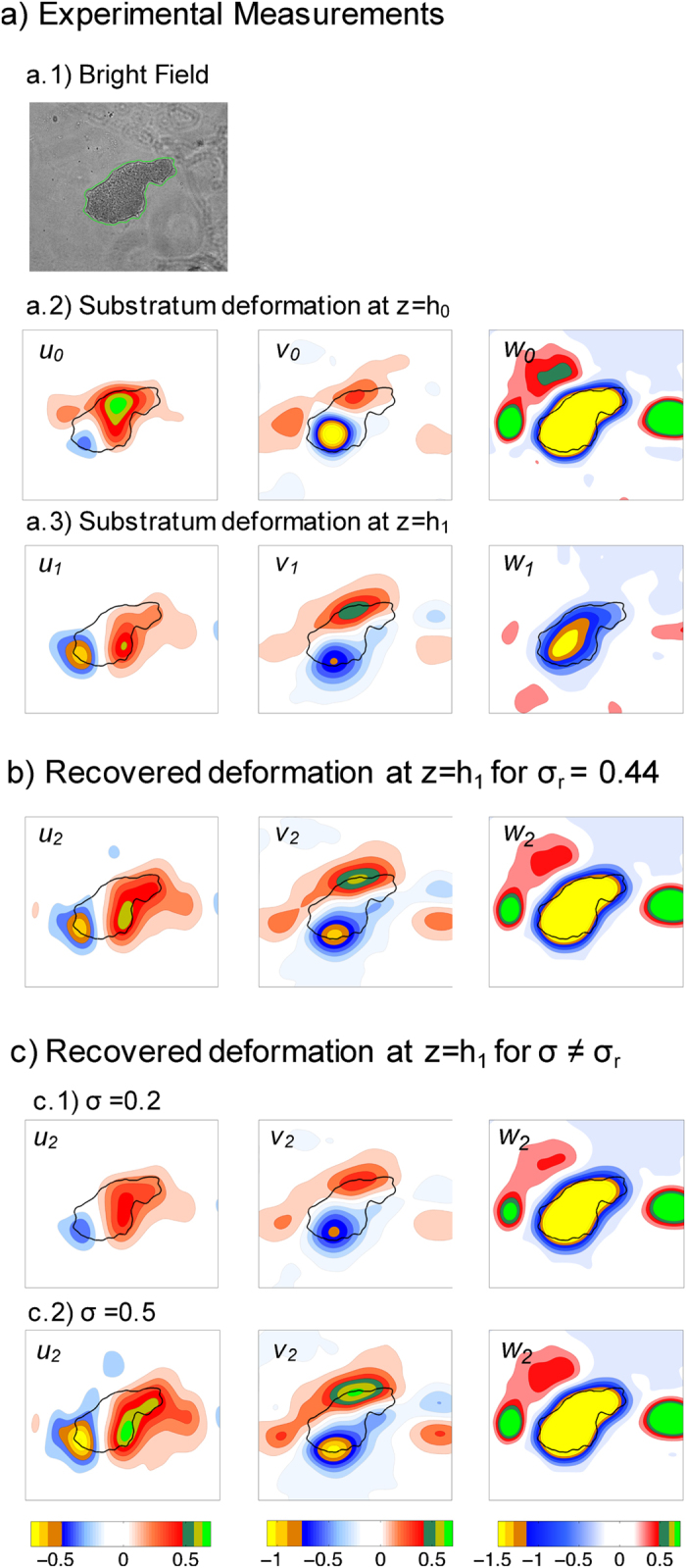
Experimental demonstration of 2LETFM. (**a**) Experimental measurements of the deformation applied by a motile *Physarum* amoebae, (a.1) bright field image of the amoeba with its detected contour plotted in green, (a.2) 3-D substratum deformation generated by the amoeba, **u**_0_, measured at the top surface of the substratum (*z* = *h*_0_ ≈ *h* = 170 *μm*), (a.3) 3-D substratum deformation generated by the amoeba, **u**_1_, measured at a second plane underneath the surface (*z* = *h*_1_ = 154 *μm*). (**b**) 3-D deformation **u**_2_ at *z* = *h*_1_ calculated from the measured **u**_0_ for the value of the Poisson’s ratio recovered by the two-layer approach, *σ*_*r*_ = 0.44. (**c**) 3-D deformation **u**_2_ at *z* = *h*_1_ calculated from the measured **u**_0_ for two values of the Poisson’s ratio below and above *σ*_*r*_. (c.1) *σ* = 0.2. (c.2) *σ* = 0.5. All the deformations are given in microns according to the colorbars at the bottom of each column.

Figure 7 shows a representative example of a two-layer deformation measurement. Panel (a) shows the experimental measurements, including the bright field image used to segment the cell contour (a.1), and the deformations measured at *z* = *h*_0_ (**u**_0_, a.2) and *z* = *h*_1_ (**u**_1_, a.3). We applied the 2LETFM algorithm to these measurements and recovered a value of *σ*_*r*_ = 0.44, which agrees well with previous measurements of the Poisson’s ratio of polyacrylamide gels^20^,^48^,^49^. As expected, the deformation **u**_2_ calculated at *z* = *h*_1_ for *σ*_*r*_ (panel b) agreed with the measured one better than for other values of *σ* ≠ *σ*_*r*_. For reference, panel (c) shows maps of **u**_2_ calculated for values of the Poisson’s ratio below (*σ* = 0.2, c.1) and above (*σ* = 0.5, c.2) the recovered value.

**Figure 8:**
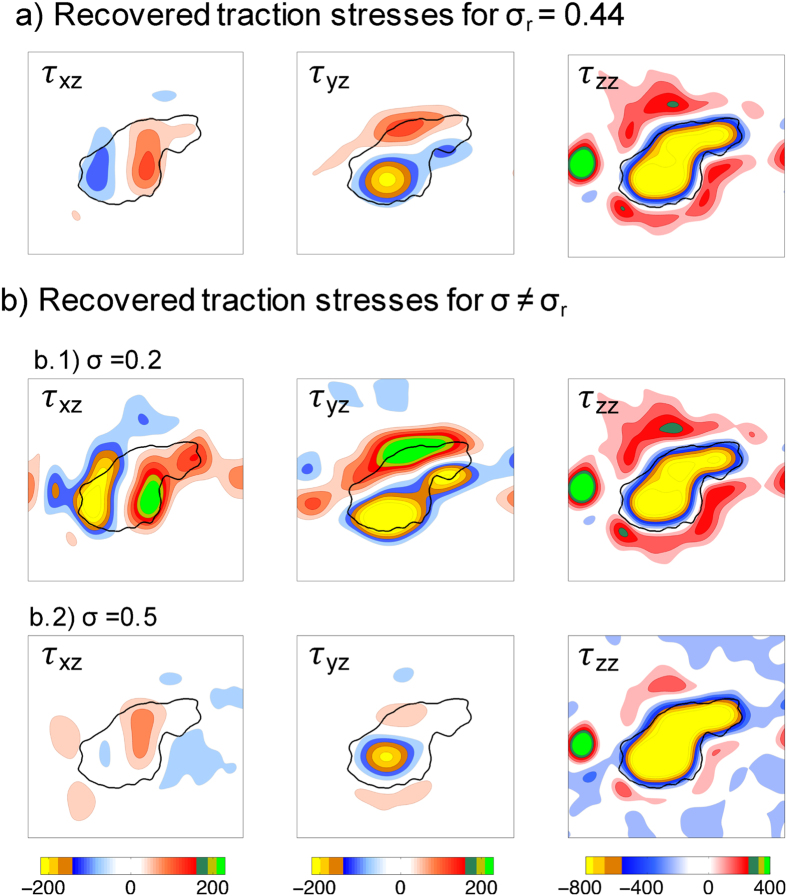
Effect of Poisson’s ratio on the 3-D traction stresses recovered from measured substratum deformation. The data comes from the same *Physarum* amoebae shown in Fig. 7. (**a**) 3-D traction stress vector (*τ*_*xz*_, *τ*_*yz*_, *τ*_*zz*_) obtained using the value of the Poisson’s ratio recovered by the two-layer approach, *σ*_*r*_ = 0.44. (**b**) 3-D traction stress vector obtained for *σ* ≠ *σ*_*r*_. The two Poisson’s ratios considered are *σ* = 0.2 (b.1) and *σ* = 0.5 (b.2). All the stresses are given in Pascals according to the colorbars at the bottom of each column.

Apart from offering potentially useful information about the material properties of the substratum, an accurate determination of *σ* is important to quantify the magnitude and spatial distribution of the traction stresses exerted by a cell. To demonstrate this point, we compared the 3-D traction stresses determined from the substratum deformations generated by the same amoeba for the actual value of the Poisson’s ratio recovered by 2LETFM, *σ*_*r*_, (Fig. 8a), and for two hypothetical values lower (*σ* = 0.2) and and higher (*σ* = 0.5) and than *σ*_*r*_ (Fig. 8b). It is important to note that since the cell is sandwiched between the substratum and an agar cap (see Materials and Methods), the computed traction stresses need not balance to zero. The results from these calculations indicate that carrying out the TFM analysis with erroneous values of the Poisson’s ratio leads to an underestimation (*σ* > *σ*_*r*_) or an overestimation (*σ* < *σ*_*r*_) of the traction stresses for the same values of measured deformations. Moreover, using inaccurate values of the Poisson’s ratio can also significantly alter the spatial patterns of the traction stress.

### Poisson’s ratio of polyacrylamide gels determined by two-layer elastographic TFM

**Figure 9:**
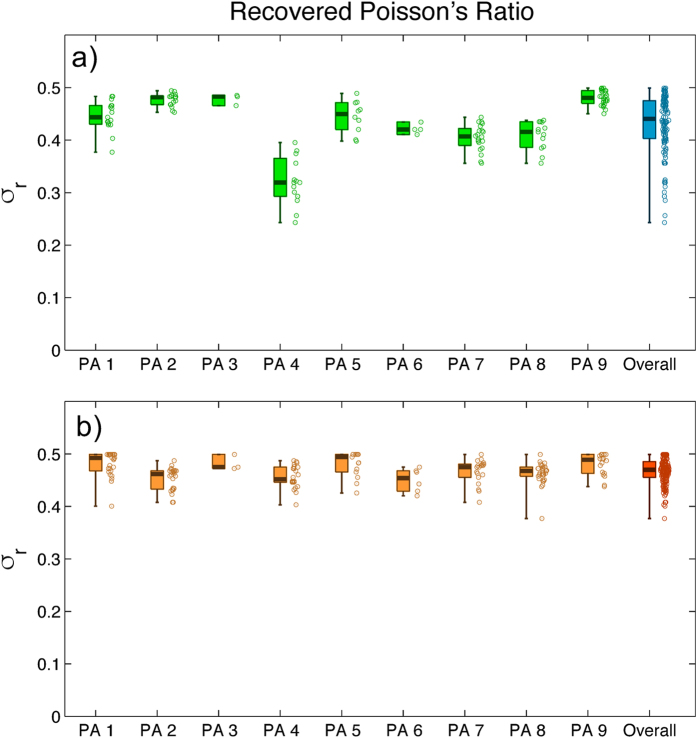
Statistics of Poisson’s ratio values determined in nine different experiments using polyacrylamide (PA) substrata. The data are presented in the form of boxplots showing the median, the lower and upper quartiles, and the minimum and maximum values of the distribution. The distribution of values for each case is represented scattered to the right of each boxplot. Each data point corresponds to an instantaneous 2LETFM measurement as the cell migrates over the substratum. Each individual substratum is represented by a boxplot, whereas all the data are pooled in the last panel at the right of the graph. The Poisson’s ratios were obtained using the cost functions *J*_1_ and *J*_2_ (eq. 5) in panels (**a**) and (**b**) respectively.

We applied 2LETFM to *Physarum* amoebae migrating over 9 different polyacrylamide gels that were manufactured using the same protocol. For each substratum, we obtained several repeated measurements of *σ* for a period long enough for the amoebae to move out of the field of view. Figure 9 shows the results of these experiments for both cost functions considered in the optimization algorithm (see eq. 5). When using the cost function *J*_1_ based on the difference between measured and calculated displacements fields, the mean ± standard deviation of the recovered Poisson’s ratio were 0.43 ± 0.05, whereas the median, first and third quartiles were respectively 0.44, 0.40 and 0.47. On the other hand, when using the cost function *J*_2_ based on cross-correlation, the mean ± standard deviation of the recovered Poisson’s ratio were 0.47 ± 0.03, whereas the median, first and third quartiles were respectively 0.47, 0.45 and 0.49. Combining the results obtained from the two different cost functions, we obtain that *σ* = 0.45 ± 0.06. The two sets of results agree with each other reasonably well, and are also in good agreement with previous direct measurements of *σ*^20^,^48^,^49^.

## Discussion

Linearly elastic polymer-based hydrogels such as polyacrylamide are broadly used as substrata to calculate the traction stresses exerted by cells. A priori knowledge of the mechanical properties of these substrata is crucial for a precise calculation of the traction stresses. In linearly elastic materials the stresses are related to the deformations by two parameters, the Young’s modulus of elasticity (*E*) and the Poisson’s ratio (*σ*). Since the traction stresses are directly proportional to *E*, significant efforts have been devoted to characterize this parameter in the context of traction force microscopy. There are well-established techniques to accurately measure *E* such as indentation, atomic force microscopy and manipulation of spherical beads^50^,^51^. On the other hand, less effort has been devoted to characterizing *σ* due to the assumption that this parameter barely influences the traction stresses. However, we recently showed that the calculation of the traction stresses exerted by cells can be highly dependent on *σ*, particularly for the nearly incompressible gels with *σ* ≈ 0.5 that are used in traction force microscopy^24^. Of note, whereas errors in *E* only affect the magnitude of the traction stresses, errors in *σ* also distort their spatial patterns (see Fig. 8). The latter may lead to qualitative misinterpretations of a traction force microscopy experiment.

This work introduces a novel two-layer elastographic 3D traction force microscopy (2LETFM) method to measure the Poisson’s ratio of an elastic substratum, while simultaneously calculating at each time point the traction stresses. The method is based on 3D measurements of substratum deformation performed on two different planes beneath the surface. The deformation measured at the first plane is used to calculate the deformation at the second plane, which is also measured. Then, it employs an optimization algorithm that recovers *σ* by iteratively minimizing a least-square cost function that penalizes differences between the calculated and measured deformations at the second plane.

We have simulated this new 2LETFM method *in silico* using synthetic 3D fields that mimic the deformation caused by large motile amoebae^36^, including random noise of different levels. By means of these simulations, we have studied the effect of several algorithmic and experimental parameters on the accuracy and robustness of 2LETFM. The results suggest that 2LETFM can accurately determine the substratum Poisson’s ratio even for noisy experimental data with signal-to-noise ratios lower than one. These simulations show that the error in the recovered Poisson’s ratio is in fact much lower than that of the recovered traction stresses, consistent with the recovery of *σ* being highly overdetermined. The method performs particularly well for *σ* ≥ 0.4, which coincides with the range of reported values of the Poisson’s ratio of hydrogels used in traction force microscopy. We found these results to be relatively independent of the cost function used in the optimization algorithm. Our *in silico* analysis with zero-mean random noise shows that a cost function based on the difference between deformations slightly over-performs a cost function based on cross-correlation. However, additional simulations (not shown) suggest that cross-correlation may be better suited to compare deformations that contain systematic errors.

We have also found that, while the position of the second measurement plane only has a modest influence on the efficacy of 2LETFM, there is an optimal inter measurement plane distance that minimizes the error of the technique. Our simulations suggest that this optimal distance is similar to the size of the spatial patterns of the deformation measured the top plane (the 1/*e* witdh *μ*, see Fig. 2). According to del Álamo *et al*.^28^, that is the characteristic distance of propagation of the cell-generated deformations into the substratum. Thus, if the second plane is located below the optimal position, the deformation measured at that plane would be very small. On the other hand, if the second plane is located above the optimal distance, the difference between the deformations measured at the two planes becomes hard to distinguish from the background noise. Consistently, the error in the recovered Poisson’s ratio increases when the cell-generated normal traction stresses are larger in magnitude than the tangential ones, because the substratum deformation changes little between the measurement planes in that case. We argued that this behavior is due to the fact that cell-generated stresses propagate deeper into the substratum when they are applied in the direction normal to the substratum surface^24^,^52^. Overall, our simulations allowed us to determine the experimental parameter values that minimize the error of the recovered *σ*, and also showed that 2LETFM can accurately and robustly recover *σ* for a wide range of experimental parameter values.

To provide proof of principle of 2LETFM, we carried out experiments with motile *Physarum* amoebae crawling on top of polyacrylamide gels, and jointly recovered the 3-D cell generated traction stresses and the gel Poisson’s ratio. Overall, these experiments resulted in *σ* = 0.45 ± 0.06, which agrees well with previously reported values of *σ* obtained by direct measuring methods^15^. For polyacrylamide gels with similar composition as in our experiments, Takigawa reported that *σ* = 0.46 by measuring the stretch ratio between the elongation of the gel in the directions perpendicular and parallel to the mechanically stretched one^48^. Also, Bodou *et al*. determined a value of 0.49 by measuring the deformation exerted through micropipette aspiration experiments^49^. More recently, Chippada measured the displacements of non-spherical magnetic inclusions moved by a magnetic manipulator to obtain a value close to 0.5^20^.

In all these methods, an external force is applied to the substratum by a relatively invasive apparatus prior to recovering the Poisson’s ratio. It is important to note that in 2LETFM, the external force is generated in the least possible disruptive manner (i.e. by the cell itself), and at the site of interest. This poses an advantage with respect to existing methods since the internal microstructure of polyacrylamide gels is not perfectly uniform^53^,^54^. The differences in the internal composition increase with the ratio of cross-linking to monomer^53^, and this inhomogeneous gel microstructure has an effect on its mechanical properties. Additionally, it has been suggested that the percentage of acrylamide and bisacrylamide and the amount of ammonium per sulfate influence the value of the Poisson’s ratio^20^,^21^.

Two-layer ETFM works by applying a mechanical equilibrium constraint on a set of over-determined substratum deformation measurements. This same principle, either using the same or a different mathematical formulation, could be exploited in a wide range of different scenarios. For example, one could adapt the current formulation to determine the 3D traction stresses generated by a cell from 2D-only measurements of the deformation in two planes. Fibrous extracellular matrices (ECMs) formed by collagen or fibrin are more physiologically relevant environments than polymer hydrogels^55^. However, the material properties of these matrices are non-linear and may also be anisotropic depending on the orientation of the fibers^56^,^57^. Extending 2LETFM to these ECMs would require reformulating the mechanical equation of equilibrium (1) to take into account large strain and anisotropy. Another potential extension of 2LETFM could be the study of the interaction between tumor cells and the ECM. Oncogenic processes commonly trigger ECM remodeling by the secretion of metalloproteinases and other ECM digestive enzymes^58^,^59^. Our method could be extended to characterize the spatial and temporal changes in the ECM in migrating cancer cells and could aid in the understanding of the involvement of the ECM in tumor development^60^,^61^.

## Additional Information

**How to cite this article**: Álvarez-González, B. *et al*. Two-Layer Elastographic 3-D Traction Force Microscopy. *Sci. Rep.* **7**, 39315; doi: 10.1038/srep39315 (2017).

**Publisher's note:** Springer Nature remains neutral with regard to jurisdictional claims in published maps and institutional affiliations.

## Supplementary Material

Supplementary Information
