# Supplementary material for: Two-Layer Elastographic 3-D Traction Force Microscopy
Source: Sci Rep. 2017 Jan 11;7:39315. doi: 10.1038/srep39315 (PMC5225457; doi:10.1038/srep39315)
Supplement: Supplementary Information [file srep39315-s1.pdf]

Two-Layer Elastographic 3-D Traction Force Microscopy: Supplementary Information

Begoña Álvarez-González[1,2], Shun Zhang[2], Manuel Gómez-González[2], Ruedi Meili[1,2], Richard A. Firtel[1], Juan C. Lasheras[2,3,4], and Juan C. del Álamo[2,4,*]

[1]Division of Cell and Developmental Biology, University of California, San Diego
[2]Department of Mechanical and Aerospace Engineering, University of California, San Diego
[3]Department of Bioengineering, University of California, San Diego
[4]Center for Medical Devices and Instrumentation, Institute for Engineering in Medicine, UC San Diego
[*]Correspondence to: jalamo@ucsd.edu

Continuity of deformations across different substratum layers

See figure S1.

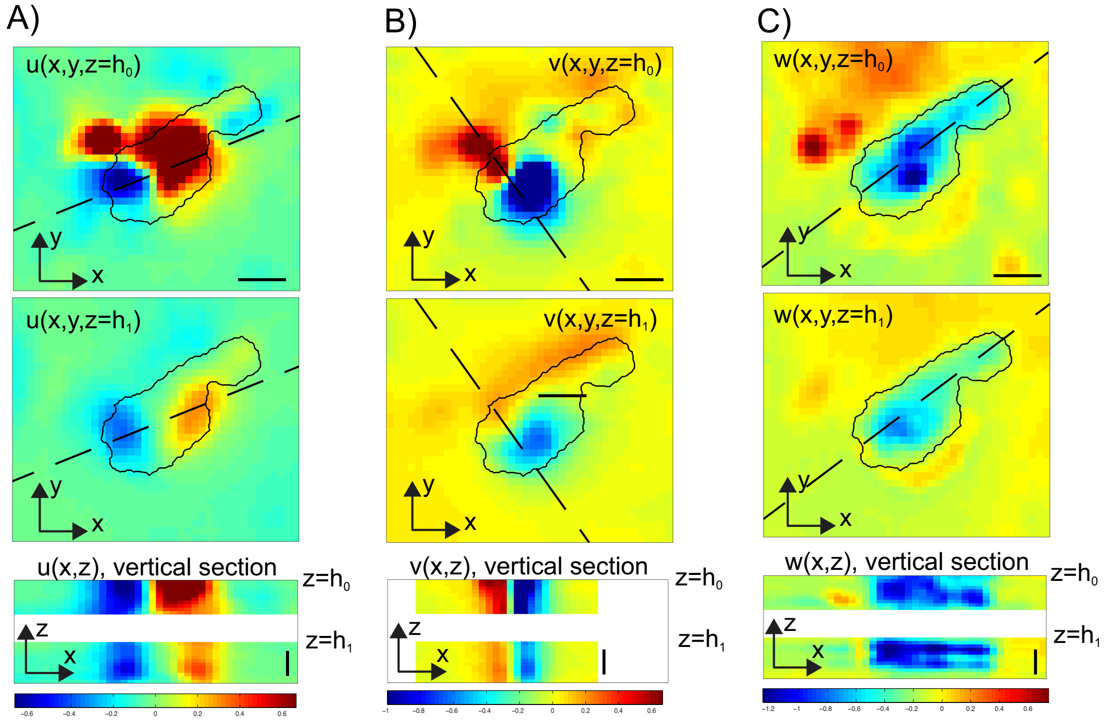

Figure S1. Continuity of deformation across polyacrylamide layers. (*a*) Top panel: Experimental measurement of the x-deformation applied by a motile *Physarum* amoeba at the top xy-plane of the substratum, $u(x,y,z=h_0)$, obtained by tracking red fluorescent beads embedded in the top substratum layer. The scale bar is 50 μm long. Center panel: Experimental measurement of the x-deformation at a second xy-plane underneath the surface, $u(x,y,z=h_1)$, obtained by tracking green fluorescent beads embedded in an intermediate substratum layer. Bottom panel: Experimental measurements of the deformation in a vertical xz-section of the substratum indicated by the dashed lines in the top and middle panels, $u(x,z)$. The deformations obtained by tracking both red and green beads in different substratum layers have been stacked to demonstrate the continuity of the measured deformations in the z direction. The scale bar is 5 μm high. (*b*) Same as (*a*) but this time the y-deformation v exerted by the cell on the substratum is represented. (*c*) Same as (*a*) but this time the z-deformation w exerted by the cell on the substratum is represented.

Traction force microscopy can be performed on a linearly elastic substratum when constant body forces and/or external contact stresses act upon the substratum

The equation of elastostatic equilibrium (1), which is solved to recover traction stresses from measurements of substratum deformation, is generally accepted to hold when both the body forces and external contact stresses acting on the substratum

are negligible. Here, we demonstrate that this equation also holds in the presence of appreciable body forces and contact stresses, as long as these are constant in time and the substratum is linearly elastic. Relaxing the assumption of negligible body forces and external contact stresses can be used to broaden the application of traction force microscopy to a wide range of non-conventional experimental setups. Particularly, it covers the setup used in our study, where the substratum is subjected to the weight of an agar cap during the duration of our microscope recordings. We still assume that the inertia and viscosity of the substratum are negligible, leading to this equation of equilibrium for the substratum at an instant of time $t_{with\,cell}$ when the cell is present in the field of view

$$\nabla \cdot [\tau_{elastic}(\mathbf{u}_{with\,cell})] + \rho \mathbf{f} + \delta(S)\tau_{contact} = 0, \qquad \text{(SI1)}$$

and to this other equation at a different instant of time $t_{whitout\,cell}$ when the cell is absent from the field of view

$$\nabla \cdot [\tau_{elastic}(\mathbf{u}_{without\,cell})] + \rho \mathbf{f} + \delta(S)\tau_{contact} = 0. \qquad \text{(SI2)}$$

In these two equations, $\mathbf{u}_{with\,cell}$ represents the deformation caused by both the body forces and the cell, whereas $\mathbf{u}_{without\,cell}$ represents the deformation caused by the body forces and external contact stresses in the absence of the cell. The elastic stress tensor in the substratum is $\tau_{elastic}$. The time-independent body force is $\mathbf{f}$ and ρ is the substratum density. The external contact stress is $\tau_{contact}$ and $\delta(S)$ is a Dirac delta that enforces the application of this stress only at surface S (i.e. $z = h$ in the case of the agar cap). We now subtract equations (SI1) and (SI2) to obtain

$$\nabla \cdot [\tau_{elastic}(\mathbf{u}_{with\,cell}) - \tau_{elastic}(\mathbf{u}_{without\,cell})] = 0, \qquad \text{(SI3)}$$

where the body force and the contact stress cancel out because they are constant in time. We now note that if the substratum is linearly elastic, equation (SI3) is equivalent to

$$\nabla \cdot [\tau_{elastic}(\mathbf{u}_{with\,cell} - \mathbf{u}_{without\,cell})] = 0, \qquad \text{(SI3)}$$

where $\mathbf{u} = \mathbf{u}_{with\,cell} - \mathbf{u}_{without\,cell}$ is the deformation measured in our experiments by performing image correlation between z-stacks acquired at $t = t_{with\,cell}$ and $t_{without\,cell}$. Furthermore, note that $\mathbf{u}$ is the deformation caused by the traction stresses generated by the cell.

Error in the recovered traction stresses as a function of noise parameters

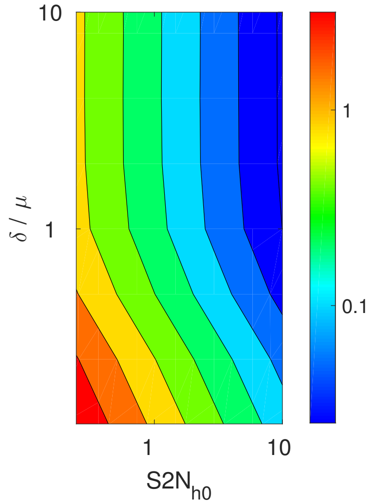

Figure S2. Relative error in the recovered traction stresses as a function of the signal-to-noise ratio of the measured deformation $S2N_0$ and the lengthscale of the noise patterns δ/μ. The data were obtained for the synthetic deformation field in eq. (6) of the main text using $\sigma = 0.45$, $h_0 - h_1 = 0.15, h_0 = 2.5\mu$ and $U_0 = W_0$.

The error is defined as

$$Err(S2N_0, \delta) = \frac{||\vec{\tau}_0 - \vec{\tau}(S2N_0, \delta)||}{||\vec{\tau}_0||},$$

where $\vec{\tau}_0$ is the ground truth traction stress vector and $\vec{\tau}(S2N_0, \delta)$ is the traction stress recovered when the deformation field has additive noise of signal-noise-ratio $S2N_0$ and lengthscale δ. This error is plotted in figure S2.

Accuracy and robustness of the recovered Poisson's ratio as a function of the noise lengthscale

See figure S3.

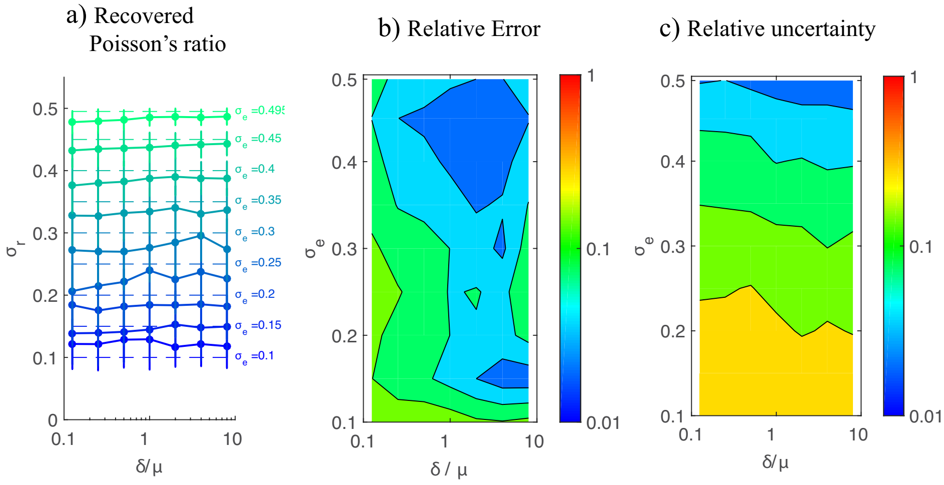

Figure S3. Accuracy and robustness of 2LETFM as a function of the lengthscale of the measurement noise, δ, normalized with the lengthscale of the deformation field at the top plane μ. (*a*) Recovered value σ_r of the Poisson's ratio, plotted as a function of δ/μ. The data are represented as mean $\pm$ standard deviation obtained from $N = 100$ random realizations. Each curve is obtained for a different value σ_e of the exact Poisson's ratio that is being recovered by 2LETFM. This exact value is indicated with a dashed horizontal line in each case. (*b*) Contour plot of the relative error of σ_r as a function of δ/μ and σ_e. The relative error is defined as $|\sigma_r - \sigma_e|/\sigma_e$. (*c*) Contour plot of the relative uncertainty of σ_r as a function of δ/μ and σ_e. The relative uncertainty is defined as r.m.s.$(\sigma_r)|/\sigma_e$. The data were obtained for $S2N = 1$ and $U_0 = W_0$.
